# Supramolecules for Pathogen Inhibition: From Polymers to Self-Assembled Nanosystems

**DOI:** 10.1021/accountsmr.5c00285

**Published:** 2026-01-28

**Authors:** Chuanxiong Nie, Christian Zoister, Guoxin Ma, Rainer Haag

**Affiliations:** † Research Building SupraFAB, Institut für Chemie und Biochemie, Freie Universität Berlin, Altensteintr. 23A, 14195 Berlin, Germany; ‡ Institut für Chemie und Biochemie, Freie Universität Berlin, Takustr. 3, 14195 Berlin, Germany

## Abstract

Vaccines and antivirals have been developed to combat virus infection, but they face the challenges of rapid and unpredictable virus mutations, which have been widely observed during COVID-19. An alternative approach is, therefore, highly needed as an additional tool to prevent virus infection. As the infection of a virus usually starts by binding to its receptor, preventing virus interaction with host cells has been considered as a promising method and has been explored by various multivalent polymeric structures. However, like small-molecule pharmaceuticals, these carefully engineered polymeric structures rarely sustain broad-spectrum efficacy, because viral proteins are morphologically diverse and evolve rapidly, enabling resistance to polymeric inhibitors through mutations in receptor-binding domains (RBDs). To address these challenges, our group developed and investigated a new class of virus inhibitors based on self-assembled supramolecules. These nanosystems are built by noncovalent conjugation of small molecules or oligomers through hydrophobic interactions, π-π stacking, hydrogen bonding, electrostatic interactions, and so on. By carefully balancing the molecular geometry and directional forces, nanostructures of different dimensions (nanofiber, nanodisk, nanosheet, nanomicelle, etc.) are obtained and functionalized with binding groups to virus spike proteins inspired by mucins, which are natural polymers forming the mucus hydrogel to prevent virus infection. By using different functional building blocks, it is possible to build heteromutlivalent nanostructures through noncovalent synthesis targeting multiple binding domains simultaneously. Distinct from covalent polymeric structures, the dynamic nature of self-assembled nanosystems allows functional groups to automatically locate complementary binding pockets on viral spike protein, thereby adapting to mutation-driven RBD changes through the adaptive presentation of binding moieties. Besides binding to virus spike protein, these nanosystems also provide steric shielding of virus particles to prevent virus interaction with host cells. These supramolecular nanosystems exhibit low toxicity and broad-spectrum antiviral activity against viruses that use distinct binding receptors, including herpes simplex virus (HSV; sulfate binding), SARS-CoV-2 (sulfate binding), and influenza A virus (IAV; sialic acid binding). To forward the application of these nanosystems, their stability should be carefully evaluated, as diverse factors in physiological conditions could affect the self-assembly of the supramolecules. Although they have been proven to be stable in cell culture conditions, a deep investigation into biological systems is still necessary. One approach to improved stability might be introducing additional reversible bonds. Besides, translating these systems will require comprehensive biosafety and bioactivity evaluation and continued chemical innovation. Collectively, these findings demonstrate the feasibility of broad-spectrum antiviral inhibitors based on supramolecular assemblies and may open new routes to design broad-spectrum virus inhibitors to assist the combat with pathogens.

## Graphical abstract

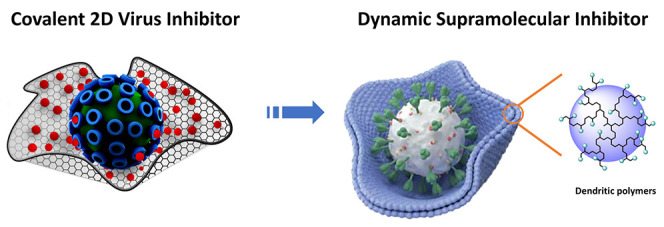

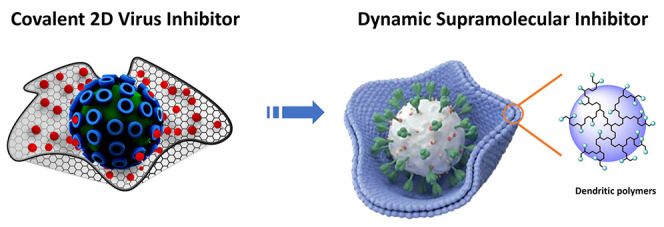

## 1 Introduction

Viral infections are a long-standing threat to public health, a reality underscored by the emergence of SARS-CoV-2.^1^ Although the global impact of COVID-19 has been mitigated by vaccines, antivirals, and public health measures, its long-term effects have not been easily diminished.^2^ SARS-CoV-2 infection has been linked to chronic, multisystem conditions that can persist for months (“long COVID”). Likewise, IAV has exerted a substantial burden: since the 1918 “Spanish flu”, IAV has circulated in humans for over a century, giving rise to thousands of subtypes and strains.^3^ The diversity of IAV and SARS-CoV-2, along with their capacity for fast mutation, complicates efforts to develop effective vaccines. Novel strategies against viral transmission are therefore needed as adjuncts when vaccine protection is incomplete or immune escape occurs.

Viral infection begins with multivalent interactions between viral surface proteins and receptors on the surface of host cells.^4^ These interactions can facilitate entry by multiple routes, including direct membrane fusion, endocytosis, and macropinocytosis. Once inside, the virus hijacks host cell metabolism to replicate. Although many small-molecule drugs target replication steps,^5^ polymeric materials offer distinct advantages as entry inhibitors.^6^ By binding to the virion surface and shielding against receptor engagement, they prevent cellular entry, leaving virions to degrade or be cleared by the immune system. A key parameter for viral entry inhibitors is their capacity to outcompete virus-cell interactions. Accordingly, polymeric entry inhibitors have been engineered with nanostructures designed to complement and engage viral spike protein, including polyglycerols,^7,8^ poly­(amidoamine) dendrimers,^9^ DNA origami,^10^ phage capsids,^11^ fullerenes,^12^ and related architectures.

Even with such tailored designs, viral mutation presents a major challenge to inhibitor effectiveness.^13^ Viral mutation during replication gives rise to unpredictable antigenic shift and drift, enabling viruses to evade vaccine-elicited antibodies. Most RNA viruses lack proofreading during their replication and therefore mutate more easily than DNA viruses—as observed during COVID-19, when hundreds of variants emerged, including several designated by the World Health Organization as variants of concern. Mutation can also render viruses fully resistant to carefully designed polymeric entry inhibitors after only a few passage cycles.^14^

One approach to this problem is to deploy highly dynamic nanosystems that can adapt to mutational change. A biomimetic perspective points to instructive analogues because virion-binding biological structures found in nature are highly dynamic. For example, mucus hydrogel is built from mucins that reversibly self-cross-link into 3D networks via dynamic hydrophobic interactions, electrostatic interactions, hydrogen bonds, and dynamic disulfide bonds.^15^ The lipid bilayers of cell membranes, where cell-surface virus receptors are displayed, are also highly dynamic. Taking inspiration from these natural systems, it should be possible to create adaptive supramolecular structures capable of binding multiple viral strains. Distinct from vaccines, the supramolecular virus entry inhibitors target the entry step of virus infection and therefore are a promising approach as an additional tool to prevent virus infection. For example, they can be formulated into nasal sprays to form a protective layer on top of the respiratory tract.

This Account summarizes recent advances in supramolecular nanosystems as viral entry inhibitors, as shown in Figure 1. Compared with conventional polymeric or nanostructured scaffolds, assemblies formed through noncovalent interactions are intrinsically dynamic and can accommodate mutation-driven changes on viral surfaces. Over the past several years, our group developed self-assembled nanosystems across dimensions, 1D nanofibers, 2D nanosheets, and 3D nanospheres, to achieve broad-spectrum virus inhibition. We built these systems from diverse building blocks, including long-alkyl-chain amphiphiles, dendritic polymers, and block amphiphilic copolymers. We characterized these systems’ self-assembly behavior, studied their virus interaction mechanisms, and quantified their inhibitory activity against multiple viruses. We also evaluated cytotoxicity and antiviral efficacy in vitro and in vivo. Therefore, with further work, it should be possible to develop potent, broad-spectrum virus inhibitors that tolerate viral mutations.

**Figure 1:**
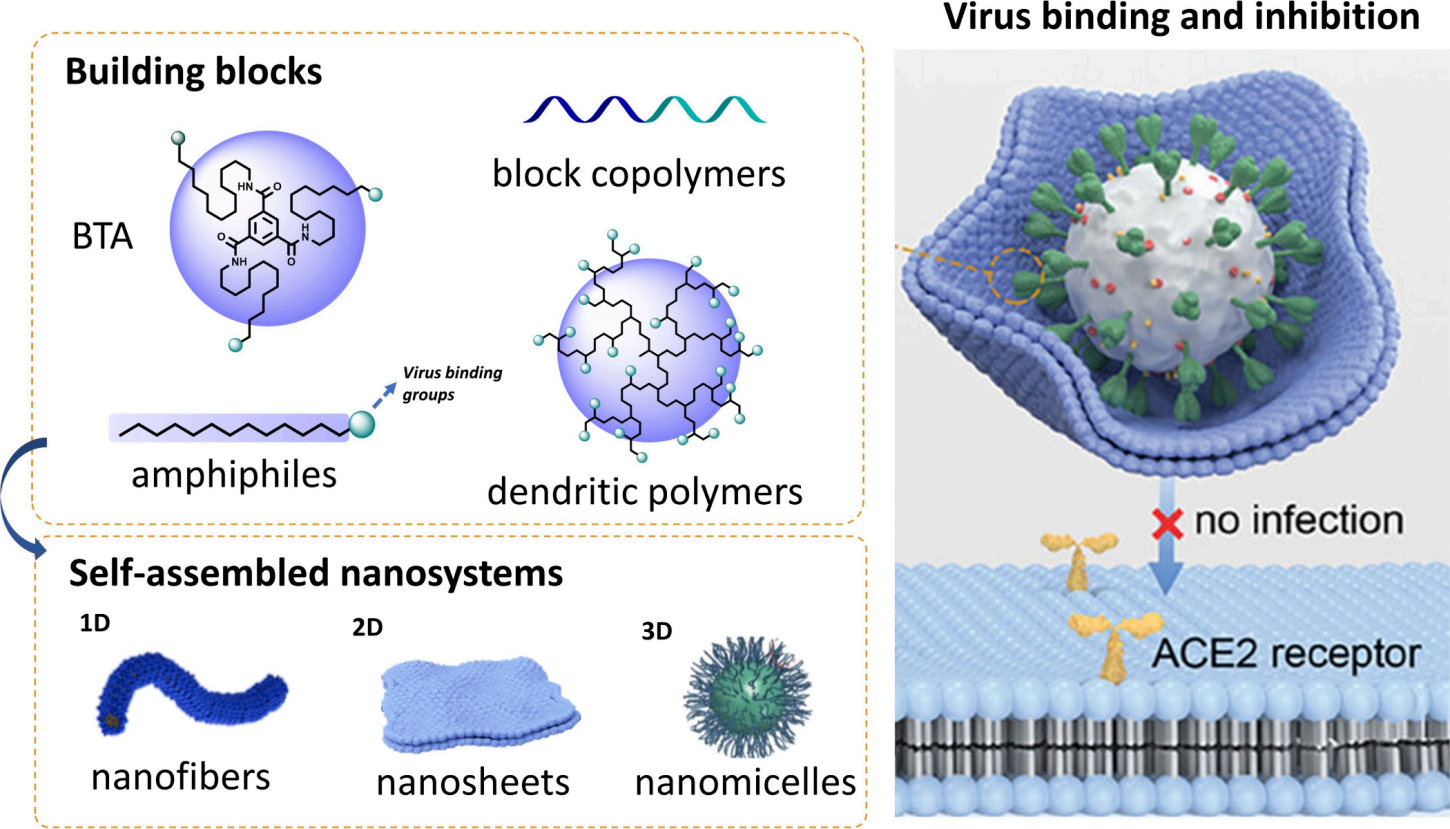
Principle of supramolecular self-assembled nanosystems for virus binding and inhibition. Supramolecular nanosystems in various dimensions (1D nanofiber, 2D nanosheet, and 3D nanomiclles) are built with various building blocks and can shield virus-cell interactions for infection inhibition. BTA: benzenetricarboxyamides. Adapted with permission from refs 16–18 . Copyrights 2024, American Chemical Society and 2024, 2025 Wiley-VCH GmbH, respectively.

## 2 Design Principles of Supramolecular Nanosystems for Infection Prevention

### 2.1 Multivalent Interactions for Virus Inhibition

To inhibit virus attachment, an entry inhibitor must bind virions more strongly than cell-surface receptors do. This competitive principle was demonstrated by Block and colleagues using quartz crystal microbalance (QCM) on supported lipid bilayers.^19^ Upon addition of excess lectins, norovirus-like particles were released from their firmly bound ligands, indicating that higher-affinity competitors can displace virions. Analogously, polymeric scaffolds engineered to outcompete the cell surface can remove virions from cells, thereby inhibiting entry. Extensive efforts on multivalent virus inhibitors have identified scaffold flexibility, size, and ligand density as the key design parameters for strain-specific potency. Multivalent structures using different polymeric back bones, such linear polymers,^7^ dendritic polymers^9^ and nanoparticles,^11^ have been designed. Linear polymers with high backbone flexibility can enable the functional groups to attach to the receptor binding domain of viruses and therefore offer higher flexibility of inhibitor design. Dendritic polymers with preorganized ligand display can attach to the virus with high affinity, but they lack the ability to cope with virus mutations. The same is also true for nanoparticle-based inhibitors. Not only are they binding to the virus, but they also generate high steric shielding effects to prevent virus interaction with host cells. Because this account focuses on supramolecular structures, readers are referred to prior reviews for comprehensive discussion of polymeric design variables.^6,20^

Multivalent interactions are the collective effect of many monovalent contacts, yielding strong yet noncovalent binding between two surfaces.^6^ In biological systems, multivalent binding underlies adhesion, recognition, and signaling. For example, glycan binding is essential for cell recognition, but single carbohydrate–protein interactions typically exhibit weak affinity with dissociation constants in the millimolar (mM) range. Multivalent presentation of glycans on the cell surface greatly increases binding strength into the low micromolar (μM), or even nanomolar (nM), range.

Viruses employ multivalent interactions with host cells, as shown in Figure 2a; one example is the interaction of IAV with sialic acid. The hemagglutinin (HA) of IAV is a trimeric protein with sialic acid-binding domains at each head. Monovalent HA-sialic acid binding is weak (K_D_ ≈ 2.8 mM),^21^ but the multivalent display of binding pockets on HA allows strong, cooperative engagement of cell-surface sialic acid during cell entry. In the influenza A/X31 (H3N2) strain, HA presents three sialic acid-binding pockets in a triangular arrangement with 5 nm spacing.^22^ This structural information has guided X31 inhibitor design: scaffolds that match the 5 nm geometry have achieved the highest binding affinity and antiviral activity.^7,9,11^ A good example is the linear polyglycerol (LPG)-sialoside conjugate (LPG-sialoside; Figure 2b,c),^7^ which exhibits an optimal 5 nm spacing between adjacent sialic acids for virus binding and infection inhibition.

**Figure 2:**
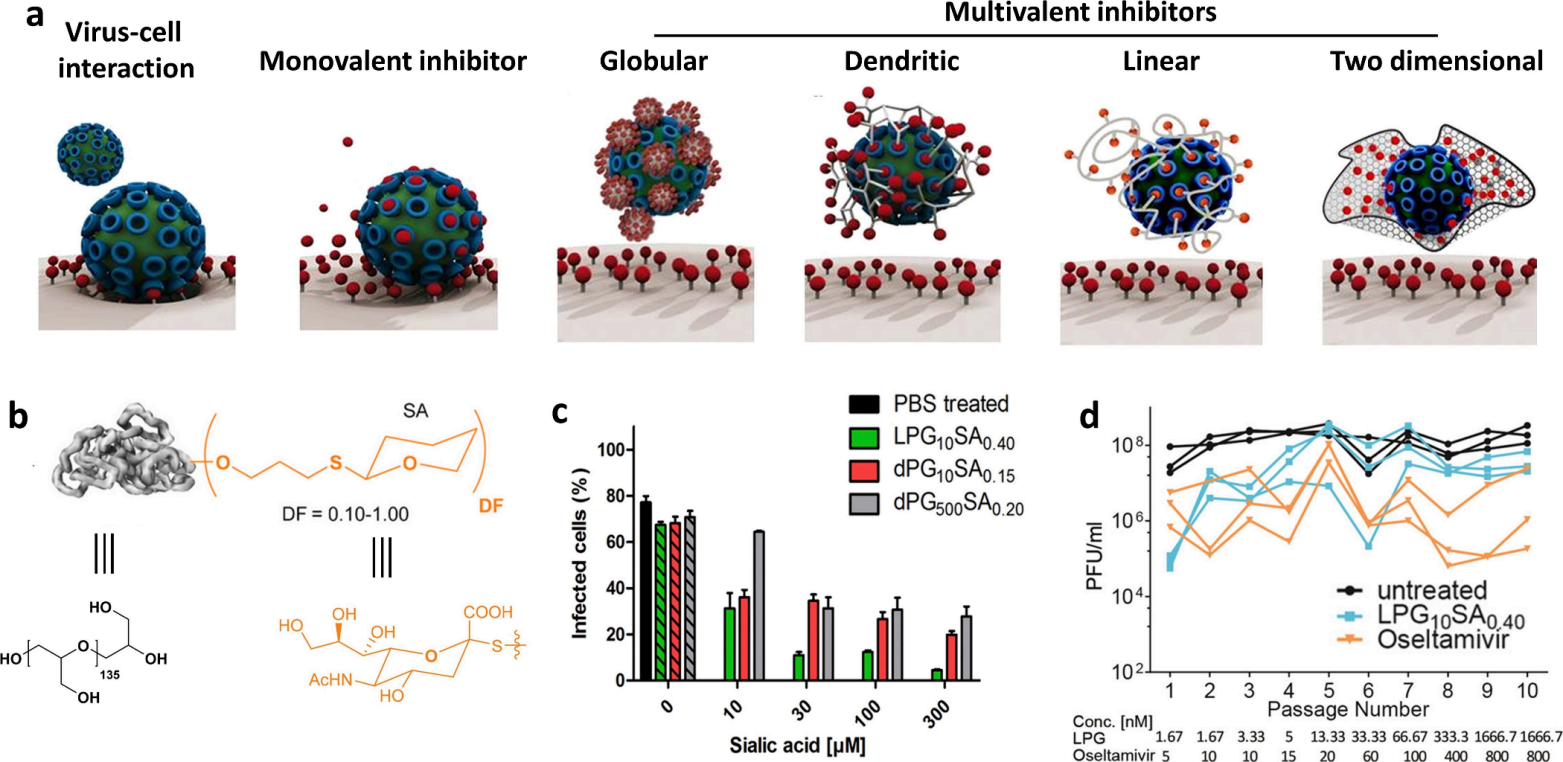
(a) Multivalent interactions underlying viral attachment; their inhibition by monovalent and multivalent inhibitors. Adapted with permission from ref 6. Copyright 2016 American Chemical Society. (b) Synthesis of PG-sialosides of different sizes and degrees of functionalization (DF). (c) Inhibition activity of LPG-sialosides with the influenza A/X31 (H3N2) virus at a MOI of 0.1. Adapted with permission from ref 7. Copyright 2017 Elsevier Ltd. (d) Serial passaging of influenza A/X31 (H3N2) with LPG_10_SA_0.40_ and oseltamivir. Adapted with permission from ref 14. Copyright 2021 American Chemical Society.

Nonetheless, viral mutations remain a major challenge. The problem is acute for RNA viruses such as IAV and SARS-CoV-2, which have high mutation rates. In a recent study from our group, IAV became resistant to a carefully designed multivalent inhibitor after only two passage cycles (Figure 2d).^14^ Increasing the inhibitor dose temporarily restored activity, but complete resistance emerged after ten passage cycles.

### 2.2 Self-Assembly of Amphiphilic Molecules into Supramolecular Nanostructures

Self-assembly is the organization of many monomeric units into predefined architectures driven by multiple weak reversible interactions between monomers.^23^ Supramolecular polymers arising from these interactions exhibit emergent properties that are not predictable from the linear sum of the monomers’ physicochemical properties.^24^ Key interactions in self-assembly include hydrogen bonding, electrostatic interactions, π–π stacking, hydrophobicity, and van der Waals forces. Each interaction is reversible and therefore imparts dynamic character; in combination, these interactions tune structural and functional properties as the system seeks a minimum in Gibbs free energy (that is, seeks thermodynamic stability).^25^

Amphiphiles with dual water affinities tend to self-assemble at interfaces or in solution to adopt preferred orientations.^26^ In nature, surfactants and phospholipids self-assemble into emulsifiers and phospholipid bilayers, respectively.^27^ Because phospholipids and most surfactants are small molecules, their low molecular weight limits chemical and physical tunability; extending the amphiphile concept to polymers enables control over chain length, defined sites for chemical functionalization, and access to more complex morphologies.^28^ By adjustment of the chain lengths of polymer blocks and their inherent hydrophilicity or hydrophobicity, one can obtain spherical micelles, cylinders, vesicles, and lamellar sheets. Our approach to supramolecular virus inhibitors across all three dimensions (1D, 2D, and 3D) is inspired by nature, where the self-assembly of amphiphilic molecules is omnipresent. Achieving assembly into specific morphologies needs a careful balance of molecular geometry and directional forces. An assembly without directional forces may result in random micelles or liposomes, while introducing 2D coplanar intramolecular interactions can lead to the formation of 2D nanosheets. For example, in our dPG-nanosheets,^17^ by introducing ionic carboxylate side groups, we can control their assembly into 2D nanosheets with different sizes. Formation of 1D nanofiber requires vertical intramolecular interactions, such as the formation of benzenetricarboxyamides (BTA) nanofibers, which is driven by the strong hydrophobic and Π-Π interactions of BTA molecules.

## 3 Self-Assembled Nanosystems for Virus Inhibition

### 3.1 1D Nanosystems

One important class of supramolecular polymers comprises synthetic 1D fibers that mimic natural supramolecular aggregates in the ECM and mucus. A well-studied example is BTAs, investigated extensively by the Meijer group.^29^ 3-fold hydrogen bonding, together with π–π stacking, drives supramolecular organization into C3-symmetric stacks.^30^ In collaboration with our group, detailed mechanistic studies revealed that C12-nBTA forms a double helical structure; hydrophobic shielding effects, comparable to those in lipid bilayers, have been proposed to explain the double helix. Other BTAs display notably different architectures. Dendritic BTA (C12-dBTA) coassembles with nBTA to produce fibers with a different helical pitch. C16-dBTA assembles as two parallel single fibers without a helical turn, yielding a stiff, highly stable morphology.^31^

Multiple BTAs have been functionalized for biomedical applications. For drug delivery, Bakker et al. demonstrated a dual strategy in which small hydrophobic molecules are encapsulated in the interior while siRNA is complexed on the exterior.^32^ An additional well-established strength of BTAs is their accessible dynamics: hydrogen–deuterium exchange coupled with mass spectrometry (HDX-MS) offers insight into the exchange of labile hydroxyl and amide protons, providing a time scale for monomer exchange with the aqueous surroundings; this time scale, in turn, correlates with fiber stiffness.^33^ Nevertheless, building supramolecular BTA-based polymers requires substantial synthetic effort to functionalize BTA monomers for specific biomedical functions. Accordingly, our recent work has focused on readily available surfactants and their roles in supramolecular coassemblies.

Together, we recently engineered BTA-surfactant coassemblies that display morphological changes and tunability typically achieved with BTA comonomers. Intercalation of chiral nonionic surfactants (dTG-C12 and DDM; Figure 3a) into the hydrophobic pocket of C12-nBTA induces asymmetry and correlates with pronounced morphological changes. As the surfactant molar ratio increases, the nBTA double helix converts to a single fiber and, at high ratios, to comicelles.^34^ Our branched surfactants provide a powerful handle to modulate and functionalize supramolecular polymers. Ongoing work by our group explores BTA-surfactant coassemblies as multivalent virus inhibitors, in which oligoglycerol surfactants bearing virus-binding receptor groups will be intercalated into BTA fibers to yield flexible, dynamic fibers. Overall, we believe that noncovalent synthesis inspired by nature may enable morphological adaptation to the continual evolutionary changes of viral pathogens.

**Figure 3:**
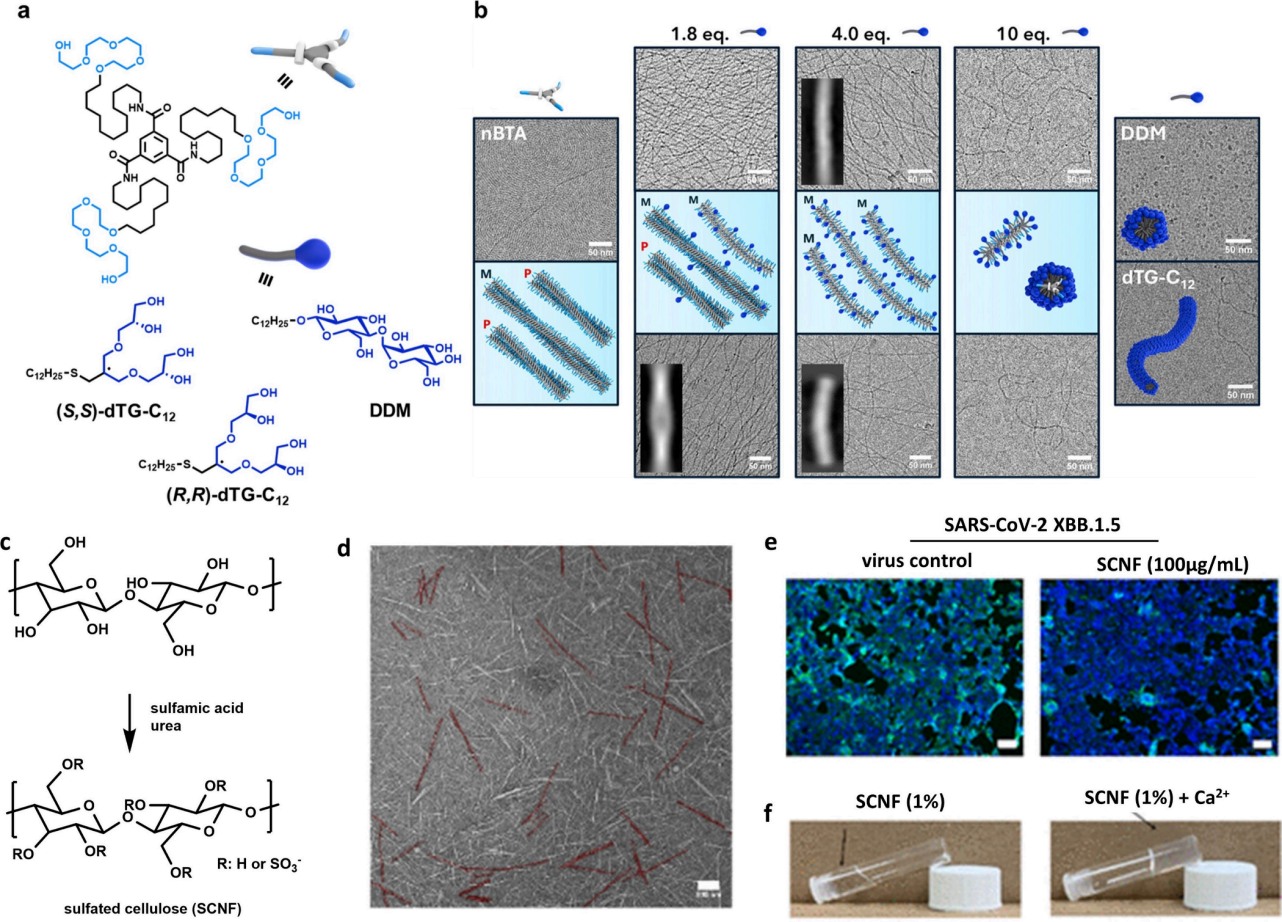
(a,b) Overview of water-soluble benzenetricarboxyamide (BTA) motifs and their self-assembly into fibrous supramolecular assemblies. (a) Molecular structures of C12-spacer BTA derivatives bearing tetraethylene glycol (nBTA) or dendritic triglycerol (dTG-BTA) substituents. (b) Visualization of nBTA fiber morphology changes induced by surfactant addition. Adapted with permission from refs 31 and 34 . Copyrights 2021, 2025, American Chemical Society, respectively. (c) Synthesis of SCNF via a deep eutectic solvent of sulfamic acid and urea. (d) TEM images of SCNF. Scale bar: 100 nm. (e) Virus inhibitory activity of SCNF against SARS-CoV-2 omicron variant (XBB.1.5). Infected cells are marked green by antibody against the SARS-CoV-2 nucleoprotein. Scale bar: 100 μm. (f) Formation of SCNF hydrogel with Ca^2+^. Adapted with permission from ref 16. Copyright 2024, American Chemical Society.

The one-dimensional nanofibers may also be prepared via a “top-down” approach. Cellulose is a natural biopolymer that self-aggregates into fibrils via noncovalent interactions. In a recent study, by using deep-eutectic solvents, our group has successfully prepared sulfated cellulose nanofibers (SCNF) with antiviral activities, as shown in Figure 3c–f.^16^ At the degree of sulfation of 0.3, SCNF was found to have comparable anti-HSV and anti-SARS-CoV-2 activities to heparin. More interestingly, the supramolecular self-assembly nature enabled the formation of SCNF hydrogel via noncovalent interactions, which allowed the control of hydrogel properties by using different metal ions. In our study, we found that mixing SCNF with Ca^2+^ resulted in mucus-like hydrogels that were able to hinder virus penetration, thereby preventing virus infection. Such activities motivate further investigation as nasal sprays for infection prevention.

### 3.2 2D Nanosystems

Beyond the 1D, our group has developed multiple 2D nanostructures that bind and inhibit viruses. Polyglycerol (PG) is a highly biocompatible polymer platform that can be functionalized for biomedical applications, including drug delivery, surface modification, and tissue engineering.^35^ We have previously functionalized polyglycerols with sulfate or sialic acid as multivalent inhibitors against HSV,^36^ SARS-CoV-2,^37^ and IAV.^7,8^ Inspired by the success of these PG-based structures, we recently developed PG-based, self-assembled 2D nanosheets capable of wrapping and shielding virions (Figure 4).^17,38^ The core polymer was synthesized from dendritic polyglycerol (dPG) and 11-mercaptoundecanoic acid (MUA) (Figure 4a).^17^ The long aliphatic chain of MUA promoted polymer aggregation in solution, while the charged head groups limited uncontrolled growth; this balance of aggregation and electrostatic repulsion led to the formation of 2D nanosheets, evidenced by strongly pH-dependent self-assembly. Under physiological conditions (pH 7.5, 150 mM ionic strength), dPG-MUA formed flexible nanosheets with an ∼250 nm lateral size. Increasing pH by 0.1 shifted assembly toward smaller nanodiscs, and further raising pH to 7.7 eliminated self-assembly, as shown in Figure 4b.

**Figure 4:**
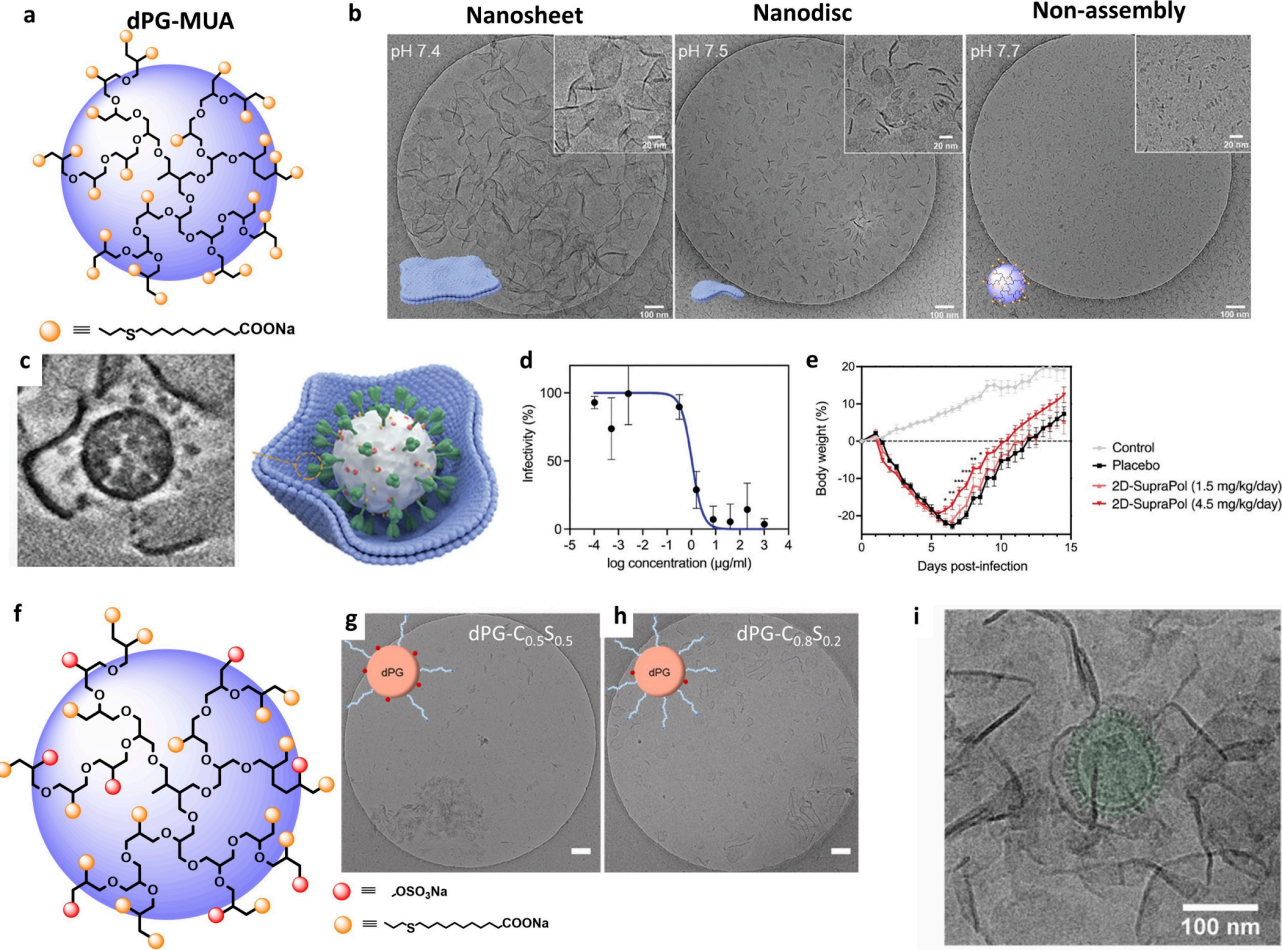
(a) Structure of dPG-MUA. The structure of dPG is abbreviated. (b) Cryo-EM images of dPG-MUA at different pH values: nanosheets (left), nanodiscs (center), and nonassembly (right) visualized by cryo-EM. Scale bar: 100 nm. (c) Interaction of SARS-CoV-2 Delta variant particles with dPG-MUA nanosheets. (d) Inhibition of SARS-CoV-2 by plaque reduction assays. Values are mean ± SD, *n* = 3. (e) *In vivo* inhibition of SARS-CoV-2 in hamsters. “2D-SupraPol” indicates dPG-MUA. Values are mean ± SD, *n* = 10. Adapted with permission from ref 17. Copyright 2024 Wiley-VCH GmbH. (f-h) Schematic morphologies of dPG with varying degrees of MUA and sulfate functionalization. dPG-C_0.5_S_0.5_ stands for dPG with 50% functionalization of MUA and 50% sulfation. (i) Interaction between influenza A virus particles and dPG-MUA nanosheets visualized by cryo-EM. Scale bar: 100 nm. Adapted with permission from ref 38. Copyright 2025 Elsevier Ltd.

This 2D nanostructure inhibited HSV, SARS-CoV-2, and IAV, which use distinct functional groups for cellular interactions.^38^ Cryo-EM images indicate that these diverse virus particles, despite differences in surface spike proteins, were wrapped by the 2D nanosheets (Figure 4c). In SARS-CoV-2 assays, the half-maximal inhibitor concentration (IC_50_) was 1 μg/mL, whereas the half-maximal cytotoxic concentration (CC_50_) exceeded 1 mg/mL, yielding a selectivity index greater than 1000. Further hamster studies proved its in vivo efficacy against SARS-CoV-2 infection. In a hamster model, a daily dose of 4.5 mg/kg accelerated recovery relative to placebo, without noticeable side effects (Figure 4e).

The self-assembly of dPG-MUA depends on the degree of MUA functionalization.^38^ We observed 2D nanosheets with functionalization above 30%, and the lateral size increased with a higher MUA content. Folding and wrinkling decreased as the MUA content rose, indicating greater nanosheet rigidity. Despite these morphological changes, dPG-MUA displayed a similar inhibitory activity against IAV across degrees of functionalization. Because IAV does not specifically recognize carboxylate groups, inhibition by dPG-MUA nanosheets appears functionalization-independent.

dPG-MUA can be further functionalized with targeting moieties to enhance virus binding. Residual hydroxyl groups on unsaturated dPG-MUA can be further modified to other functional groups; we converted them to sulfates using SO_3_•Py, which increased HSV inhibition (Figure 4f). However, inhibitory effect was not linear with the degree of sulfation; the most potent inhibitor contained 80% MUA and 20% sulfation. Increasing sulfation above 50% reduced dPG-MUA nanosheet assembly, possibly because the sulfates’ added negative charges increased electrostatic repulsion between dPG-MUA polymer chains, consistent with the morphologies in Figure 4g,h. At 20% sulfation, nanosheets still formed; the superior HSV-1 inhibition at this sulfation level underscores the advantages of self-assembled 2D nanosheets over single polymers.

### 3.3 3D Nanosystems

HSV-1 virions are enveloped spherical assemblies with diameters of 150–240 nm.^39^ Spherical inhibitors in similar size ranges have been widely studied.^40^ Many such assemblies present effective binding sites and can deform upon adhesion to virions, improving interfacial contact.^41^ In our prior work, highly sulfated linear polyglycerol (lPGS), a heparin mimic, potently inhibited HSV-1 and SARS-CoV-2.^37^ Motivated by these results, we assembled nanospheres from various lPGS chains. Nanoinhibitors near 100 nm in diameter exhibit longer circulation lifetimes than single polymer chains (∼9 nm).^42^ Accordingly, we synthesized amphiphilic block copolymers of lPGS and poly­(trimethylene carbonate) (PTMC), which self-assembled in aqueous media into core–shell micelles driven by hydrophobic interactions among the PTMC blocks. More interestingly, the lPGS polysulfate segments protruded from the PTMC core to form a hairy corona. This soft, flexible corona engaged viral surfaces and accommodated their topography and deformation, thereby shielding them against virus-host cell interactions.

As shown in Figure 5, we prepared a library of lPGS-PTMC block copolymers spanning various sulfation ratios. All formulations formed core–shell micelles with a hairy corona, but the particle size, charge density, and brush length varied. As sulfation increased from 23% to 100%, the micelles’ diameter decreased from 150 to 79 nm, while corona charge density increased. We evaluated the inhibitory activity against HSV-1 across this series. In plaque reduction assays (Figure 5), 23%-sulfated micelles showed no inhibition even at high concentrations, whereas 45%, 76%, and 100%-sulfated micelles exhibited very low IC_50_ values of 0.43 μg/mL, 0.16 μg/mL and 0.037 μg/mL, respectively. These data indicate a threshold sulfation degree near 45% and a nonlinear decrease in IC_50_ with increasing sulfation. These trends suggest that inhibitory activity derives not only from the number of sulfates but also from micelle size, hydrophilicity, and flexibility. Cryo-EM revealed the dynamic interactions between the hairy micelles and HSV-1: micelles were distributed homogeneously around virions, consistent with interparticle electrostatic repulsion, and the inner layer of micelles adhered to the viral envelope.

**Figure 5:**
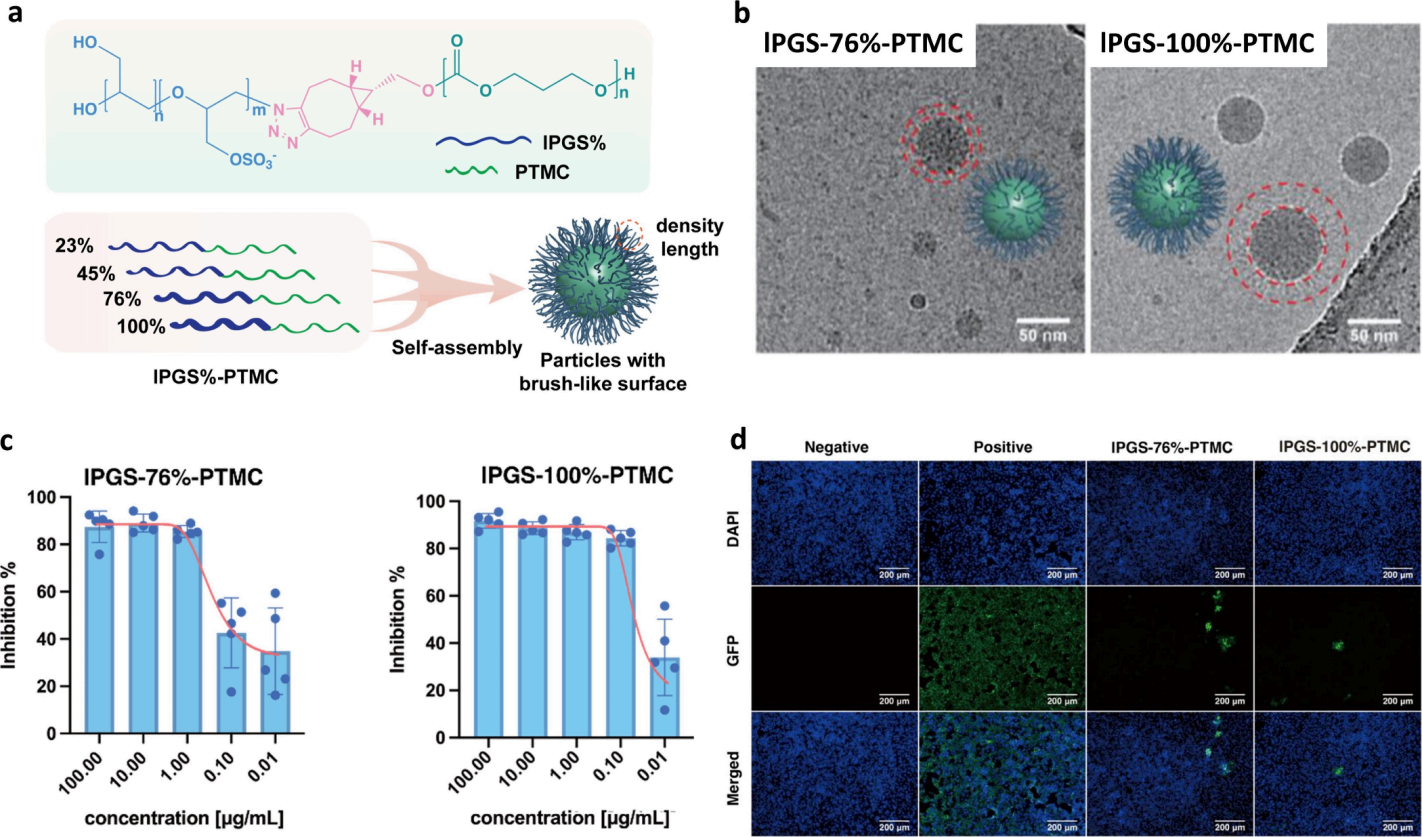
(a) Self-assembly of lPGS%-PTMC into nanoaggregates with a brush-like corona. (b) Cryo-EM images of lPGS%-PTMC nanoassemblies. Scale bar: 50 nm. (c) Dose-dependent inhibition of HSV-1 by lPGS-76%-PTMC and lPGS-100%-PTMC in plaque reduction assays. Values are the mean ± SD, *n* = 5. (d) Representative fluorescence images of Vero E6 cells infected by omicron BA.5 after preinhibition by lPGS-76%-PTMC and lPGS-98%-PTMC at 100 μg mL^–1^. Adapted with permission from ref 42. Copyright 2025 Wiley-VCH GmbH.

These highly sulfated hairy micelles exhibited potent inhibition against HSV-1, including activity after the first infection cycle. Because the RBD of the SARS-Cov-2 spike protein is positively charged, we further evaluated these micelles against the BA.5 variant. The 76% and 100%-sulfated micelles prevented infection in the majority of cells (Figure 5d). Consistent with the HSV-1 results, the 100%-sulfated micelles were slightly more potent than the 76%-sulfated micelles. Despite differences in size and flexibility, the data indicate that charge density is the primary criterion for inhibitory activity, supporting the use of these sulfated micelles as inhibitors against distinct groups of enveloped viruses.

## 4 Applications

Unlike small-molecule pharmaceuticals, polymeric and supramolecular nanostructures are always highly polydisperse. This trait complicates pharmacokinetic analysis and has hindered translation to in vivo applications. For example, the biodistribution of a nanoparticle is highly dependent on its size. Intravenously administered particles may undergo renal clearance or hepatic uptake and degradation, depending on particle size.^43^ Because reliably producing monodisperse nanoparticles at a scale remains challenging, many efforts have focused on topical or ex-vivo applications, where strict monodispersity is less critical.

One potential application of these nanosystems is formulation as a nasal spray to reduce the respiratory tract infection risk. An existing example is Algovir spray, which uses Carragelose (iota-carrageenan) as the antiviral polymer and has been shown in vitro to inhibit a wide range of sulfate-binding viruses, including herpes simplex virus, respiratory syncytial virus, and SARS-CoV-2.^44^ When delivered to the respiratory tract, it may also bond with mucus and enhance the trapping and clearance of virions. Furthermore, Astodrimer sodium, a synthetic dendritic polymer bearing naphthalene disulfonic acid functional groups, exhibits broad-spectrum antiviral and antibacterial activity.^45^ A commercial product, VivaGel, a 3% astodrimer sodium formulation in Carbopol gel, has been developed to inhibit sexually transmitted infections caused by bacteria, HSV, and HIV. Following clinical safety evaluation, VivaGel received U.S. Food and Drug Administration (FDA) Fast Track designation in 2006 for HIV prevention, and in 2015 it received approval in Europe for the treatment of bacterial vaginosis. Related products, such as VivaGel-coated condoms, have been approved for marketing in Japan, Australia, Canada, and Europe.

For the successful translation of supramolecular nanosystems, several challenges must be addressed. First, the self-assembly and stability of these materials under complex physiological conditions require more detailed studies. Because these architectures are held together by noncovalent interactions, the effects of tissue microenvironments, including salt, small molecules, and pH, should be investigated systematically. Small changes in solution conditions have produced distinct structures and biological activities in our in vitro experiments, underscoring the need to evaluate media that better reflect in vivo environments. Second, large-scale manufacturing under good manufacturing practice (GMP) conditions will demand innovation in precursor synthesis. For example, for dPG amphiphiles bearing aliphatic chains, we developed a one-pot strategy that enables multigram synthesis; with modest modifications, kilogram-scale production of dPG-MUA appears feasible.^46^ Third, the bioactivities of these supramolecular nanosystems must be characterized more rigorously. In our hamster study of dPG-MUA, we observed accelerated recovery after infection, but the data set remains limited and preliminary. More comprehensive assessments, ideally in models closer to humans, are needed to establish the efficacy and safety. To this end, we are also designing advanced ex-vivo platforms, such as a lung-on-a-chip model, to evaluate biological responses to these supramolecular nanosystems.

## 5 Conclusion and Outlook

This Account summarizes recent efforts to develop novel virus inhibitors based on supramolecular self-assembled nanosystems motivated by the need to overcome mutations through inhibitor design. Polymeric materials can be effective against specific strains when carefully engineered, but viruses readily acquire resistance through mutations in the receptor-binding domain. The dynamic nature of supramolecular self-assemblies is being explored as a solution and has proven effective in our recent work with 1D, 2D, and 3D assemblies. In particular, dendritic polyglycerol-based 2D supramolecular nanosheets have shown broad-spectrum activity against diverse viruses and have demonstrated in vivo efficacy in a hamster model. These successes motivate further exploration along these lines.

One of the biggest challenges for dynamic supramolecular nanosystems is to achieve high stability under physiological conditions, where they are normally with high salt concentrations, especially for the structures built by electrostatic interactions. In our studies of 2D dPG nanosheets, we already noticed different morphologies at different pH values. This challenge may be overcome by introducing additional interactions into the nanosystems to enhance their stability. Moreover, translation of supramolecular nanosystems faces challenges related to biodistribution and bioactivity. Examples of biopolymer-based gels, creams, and nasal sprays have motivated our investigation of supramolecular structures for topical and ex-vivo applications. However, successful clinical translation will require more comprehensive studies of the stability and bioactivity in complete biological models. Innovations in monomer synthesis will also be necessary to enable the large-scale, homogeneous production of these structures.
